# Antibiotic-induced microbiome depletion promotes intestinal colonization by *Campylobacter jejuni* in mice

**DOI:** 10.1186/s12866-024-03313-5

**Published:** 2024-05-09

**Authors:** Haohao Chen, Yanfang Zhang, Yi Pan, Lin Wu, Wenqian Wang, Hui Zhang, Hongqiang Lou

**Affiliations:** 1 Medical Molecular Biology Laboratory, School of Medicine, Jinhua Polytechnic, No. 1188 Wuzhou Street, Wucheng District, Jinhua, Zhejiang Province P.R. China; 2 Animal Center, Jinhua Food and Drug Inspection and Testing Research Institute, Jinhua, Zhejiang Province P.R. China

**Keywords:** *Campylobacter jejuni*, Colonization, TaqMan qPCR, 16S rDNA analysis

## Abstract

**Background:**

To establish a method to induce *Campylobacter jejuni* colonization in the intestines of C57BL/6 mice through antibiotic-induced microbiome depletion.

**Results:**

Fifty-four female C57BL/6 mice were divided into the normal, control, and experimental groups. The experimental group was administered intragastric cefoperazone sodium and sulbactam sodium (50 mg/mL) for 2 days; then, the experimental and control mice were intragastrically administered 200 µL *C. jejuni*, which was repeated once more after 2 days. Animal feces were collected, and the *HipO* gene of *C. jejuni* was detected using TaqMan qPCR from day 1 to day 14 after modeling completion. Immunofluorescence was used to detect intestinal *C. jejuni* colonization on day 14, and pathological changes were observed using hematoxylin and eosin staining. Additionally, 16S rDNA analyses of the intestinal contents were conducted on day 14. In the experimental group, *C. jejuni* was detected in the feces from days 1 to 14 on TaqMan qPCR, and immunofluorescence-labeled *C. jejuni* were visibly discernable in the intestinal lumen. The intestinal mucosa was generally intact and showed no significant inflammatory-cell infiltration. Diversity analysis of the colonic microbiota showed significant inter-group differences. In the experimental group, the composition of the colonic microbiota differed from that in the other 2 groups at the phylum level, and was characterized by a higher proportion of Bacteroidetes and a lower proportion of Firmicutes.

**Conclusions:**

Microbiome depletion induced by cefoperazone sodium and sulbactam sodium could promote long-term colonization of *C. jejuni* in the intestines of mice.

**Supplementary Information:**

The online version contains supplementary material available at 10.1186/s12866-024-03313-5.

## Background

Guillain-Barré syndrome (GBS) is a rare, self-limiting, post-infectious, autoimmune neurological disorder that is most commonly triggered by *Campylobacter jejuni* enteritis. The association between *C. jejuni* and GBS was first reported in 1982, by Rhodes and Tattersfield [1]. In 1991, McKhann et al. [2] reported cases of acute paralysis in young adults and children in northern China, which were later identified as axonal GBS [3]. Li et al. successfully isolated *C. jejuni* from the feces of GBS patients in China, and demonstrated that the fecal isolates induced peripheral nerve injury in a chicken model [4]. Since then, researchers have reproduced this type of *C. jejuni*-induced neuropathological damage in rabbits [5] and guinea pigs [6]. However, the use of these animal models has its limitations, such as high cost, poor reproducibility, and insufficient immune and genetic tools for an in-depth study [7]. Mice, which are commonly used animal models of infection, have not been widely used for *C. jejuni*-related models. Studies have shown that the intestinal microbiota of mice, particularly *Bacteroides*, *Clostridium*, and *Lactobacillus*, competitively prevent *C. jejuni* colonization [8, 9].

In recent years, antibiotic-induced microbiome depletion has emerged as another method for studying the role of the gut microbiota in pathological conditions, and this method can supplement the use of germ-free mice [10–12]. It is generally believed that the commensal microbiota in the animal gut can prevent the invasion of exogenous pathogens, and is an important part of the host defense mechanism [13]. The intestinal microbiota resists pathogens by consuming the nutrients necessary for the survival of the pathogens, producing bacteriocins and organic acids, changing the pH value in the intestinal lumen, or utilizing the limited oxygen in the intestinal tract [14, 15]. Antibiotics disrupt the balance of the gut microbiota and lead to bacterial lysis, which results in the release of carbon sources and elevation of bile acid levels [16]. In this study, cefoperazone sodium and sulbactam sodium were used to induce intestinal microbiome depletion in C57BL/6 mice, and a method was developed to colonize the intestines of these mice with *C. jejuni*. We hope that the creation of mouse models of *C. jejuni* enteritis will enable further research on this bacterial infection and its associated neuropathy.

## Methods

### Animals

We obtained 54 specific pathogen-free female C57BL/6 mice from Hangzhou Medical College [SCXK (Zhe) 2019-0002]. The mice were 10 weeks of age and had an average body weight of 17.57 ± 0.12 g; in addition, none of the mice had given birth. The animals were housed in the Animal Center of Jinhua Institute of Food and Drug Inspection and Testing [SYXK (Zhe) 2021-0009]. The mice were provided with sterilized drinking water and food, and maintained on a 12 h/12 h day and night cycle, with constant humidity and temperature controlled between 22℃ and 25℃. Throughout the experiment, mice that exhibited extreme distress or weight loss of over 15% of their body weight were euthanized. Ethical approval for the animal experiments was obtained from the Experimental Animal Ethics Committee of Jinhua Food and Drug Inspection and Testing Research Institute (approval no.: AL-JSYJ202103).

### Bacterial strain

*C. jejuni* (CICC22936, ATCC33291) was provided by the China Industrial Microbial Culture Collection Center and inoculated onto Columbia blood agar plates. The cultures were grown for 48 h at 42℃ under microaerophilic conditions (MGC AnaeroPack-MicroAero, Mitsubishi Gas Chemical Company Inc., Tokyo Japan).

### Animal model construction

The 54 mice were randomly divided into the normal, control, and experimental groups (18 mice/group). After 7 days of adaptive feeding, the mice in the experimental group received 200 µL of a cefoperazone sodium and sulbactam sodium solution (50 mg/mL; Pfizer Inc., New York, NY, USA) via oral gavage, which was repeated on the second day. The mice in the control group were given 200 µL normal saline, while the mice in the normal group did not receive any treatment. On the third day, the mice in the experimental and control groups were administered 200 µL normal saline solution containing *C. jejuni* (4.0 McFarland turbidity, approximately 1.2 × 10^9^ CFU/mL); this was repeated after 2 days to complete the modeling [12, 17]. In all groups, the body weight of the animals was measured before modeling, on the day of modeling completion, and at 1 week and 2 weeks after modeling completion.

### Detection of fecal *C. Jejuni* by the qPCR probe method

Fresh feces from the mice were collected daily from days 1 to 14 after modeling, and fecal bacterial DNA was isolated and purified using the QIAamp® FAST DNA Stool Mini Kit (Qiagen, Hilden, Germany), according to the manufacturer’s instructions. To 5 µL of DNA (template), we added 0.5 µL of 10 µM *hipO* primers (forward 5ʹ-GAATTTGATACCTTAAGTGCAGC-3ʹ and reverse 5ʹ-AGGCACGCCTAAACTATAGCT-3ʹ, Shanghai Sangon Bioengineering Co. Ltd., Shanghai, PRC) and probes (FAM-CTCCTTGCTCATCTTTAGGATAAATTCTTTCAC-TAMRA, Shanghai Sangon Bioengineering Co. Ltd.), 10 µL of 2× iTaq Universal Probes Supermix (Bio-Rad, Hercules, CA, USA), and 3.5 µL of ddH_2_O. Quantitative polymerase chain reaction (qPCR) assays were performed on a Bio-Rad CFX96 PCR instrument, and the thermocycling conditions were as follows: initial denaturation at 94 °C for 3 min, and 40 cycles at 93 °C for 30 s and 55 °C for 45 s.

### Histological examination

On the 7th and 14th day after modeling, animals were anesthetized with pentobarbital (5 mg/100 g, intraperitoneal injection), and sacrificed using the cervical vertebra dislocation method, according to Guide for the Care and Use of Laboratory Animals: Eighth Edition. The colon tissues were collected from the animals, fixed with a 4% paraformaldehyde solution, dehydrated in a gradient alcohol series, paraffin embedded, cut into 4-µm sections, dewaxed, and subjected to antigen retrieval with sodium citrate buffer. The sections were then permeabilized with TritonX-100 and incubated in a wet box with a blocking solution at 37 °C for 30 min. The primary antibody (rabbit anti-*C. jejuni*, 1:100, Bio-Rad) was added, and the sections were incubated overnight at 4 °C. This was followed by incubation with the secondary antibody, donkey anti-rabbit IgG H&L Alexa Fluor® 647 (dilution, 1:200; Abcam, Cambridge, UK) at 37 °C for 1 h. The sections were then stained with DAPI, and mounted with an antifade mounting medium (Beyotime Biotech Inc., Shanghai, PRC). Fluorescence microscopy images were captured (BX53, Olympus Corporation, Tokyo, Japan). In addition, some paraffin-embedded colon tissue sections were subjected to staining with hematoxylin for 5 min and eosin for 2 min, and then photographed.

### Analysis of intestinal bacterial diversity in mice

At 72 h after cefoperazone sodium and sulbactam sodium solution gavage (*n* = 3 mice/group) and at 14 days after modeling completion (*n* = 12 mice/group), the proximal colon was harvested from the mice in the 3 groups immediately after the animals were sacrificed. The colonic contents were then collected and purified using QIAamp® FAST DNA Stool Mini Kit (Qiagen), according to the manufacturer’s instructions. The purified DNA was sent to Shanghai Tianhao Biotech Co. Ltd. (Shanghai, PRC) for the identification of bacterial species by using the 16S rDNA method.

### 16S rDNA sequencing

Genomic DNA was extracted from the mouse fecal samples (QIAamp® FAST DNA Stool Mini Kit, Qiagen). The purity and concentration of the extracted DNA were measured using a Nanodrop spectrophotometer (NanoDrop One/OneC, Thermo Fisher Scientific, Waltham, MA, USA), and its integrity was assessed using 0.8% agarose gel electrophoresis. The V3-V4 hypervariable region of the 16S rRNA gene was amplified using the primers 5ʹ-CCTACGGGNGGCWGCAG-3ʹ (forward) and 5ʹ-GACTACHVGGGTATCTAATCC-3ʹ (reverse). Each DNA sample was independently amplified 3 times. The PCR products were examined using agarose gel electrophoresis, and the PCR products from the same sample were pooled to serve as a template. Index PCR was performed using index primers to add the Illumina index to the library. The PCR products were purified using the Agencourt AMPure XP Kit (Beckman Coulter, Pasadena, CA, USA). The purified products were indexed in the 16S V3-V4 library. Quality testing of the library was performed on a Qubit@2.0 fluorometer (Thermo Fisher Scientific) and an Agilent Bioanalyzer 2100 system (Agilent Technologies, Santa Clara, CA, USA). The pooled library was sequenced (Illumina HiSeq 250 sequencer) to generate 2 × 250-bp paired-end reads.

### Bioinformatics and statistical analyses

To obtain clean reads for bioinformatics analysis, we quality-filtered and merged the raw reads by using the following criteria: (1) Raw reads at any site with an average quality score of < 20 were truncated, while reads contaminated by the adapter and those with a length of < 100 bp were removed using TrimGalore. (2) Paired-end reads were merged into tags by using Fast Length Adjustment of Short Reads (FLASH, *v*1.2.11). (3) Reads with ambiguous bases (i.e., N bases) and those with homopolymers measuring > 6 bp were removed using Mothur. (4) Finally, reads with low complexity were removed.

The remaining unique reads were evaluated for chimeras by comparison with the Genomes OnLine Database (http://drive5.com/uchime/gold.fa). After the removal of the chimeras, the remaining reads were clustered into operational taxonomic units (OTUs) by using UPARSE (similarity cutoff, 97%). The OTUs were classified using the Ribosomal Database Project (RDP) Release9 201,203 (Mothur). Rarefaction analysis and analysis of alpha diversity (using the Shannon, Simpson, and InvSimpson indexes) were accomplished using Mothur. The OTUs were clustered using sample tree clustering based on the Bray-Curtis distance matrix, the unweighted pair-group method with arithmetic means, and Jaccard principal coordinate analysis. All clustering methods were performed using the R Project (Vegan package, *v*3.3.1). Redundancy analysis was conducted using Canoco for Windows *v*4.5 (Microcomputer Power, Ithaca, NY, USA), and assessed using the Monte Carlo permutation procedure (499 random permutations).

### Statistical analysis

All data were expressed as mean ± standard deviation ($\overline{x}$ ± s), and one-way analysis of variance followed by the Tukey post hoc test was used to determine statistical significance. *P*-values of < 0.05 were considered to indicate statistical significance.

## Results

### Detection of *C. Jejuni* in feces and changes in body weight of mice

**Fig. 1:**
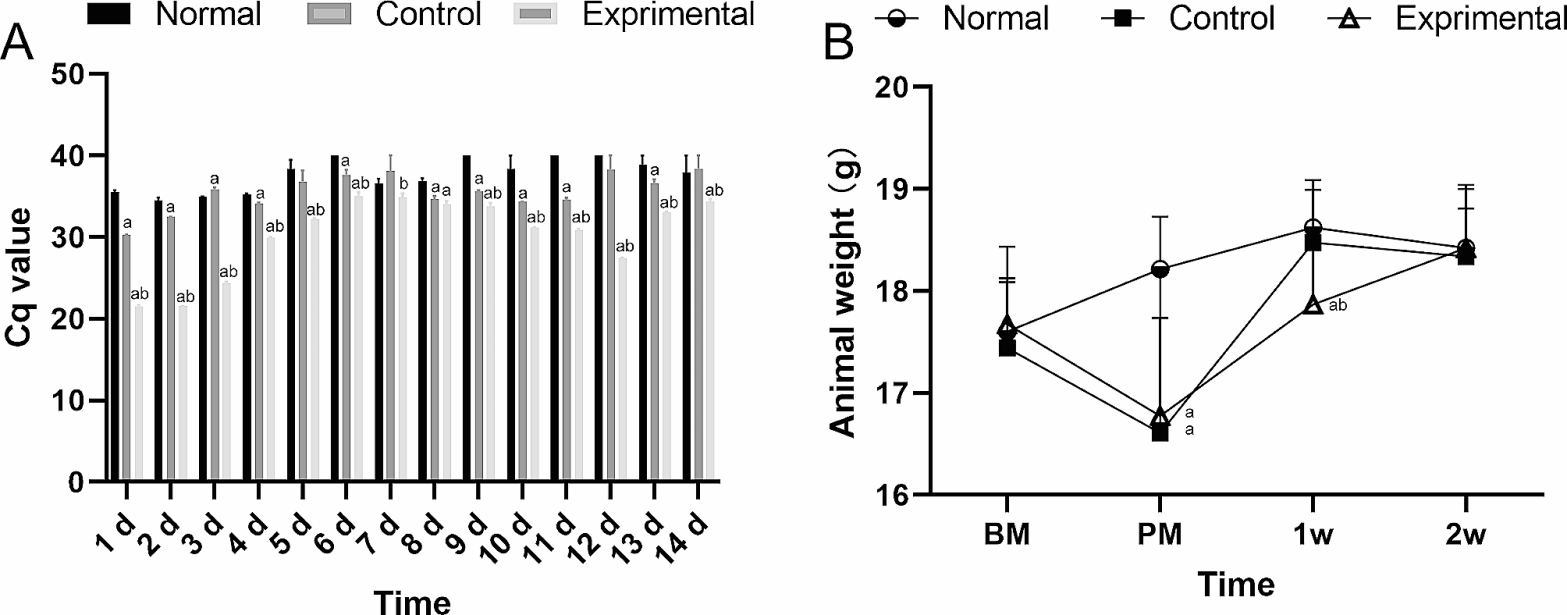
Detection of *Campylobacter jejuni* in feces and changes in body weight in the 3 groups of mice.^a^ indicates significant difference compared with the normal group. ^b^ indicates significant difference compared with the control group

Following modeling, mouse fecal samples were collected for 14 days and analyzed for the presence of the *hipO* gene of *C. jejuni* by using qPCR assays with probes. The qPCR results indicated that *C. jejuni* was detected in the feces of the animals in the experimental group from day 1 to day 14 after modeling. In contrast, the feces of the animals in the control group were only examined on days 1 and 2 (Fig. 1A). On the day of modeling completion, the body weight of the animals decreased in the control and experimental groups, but not in the normal group. At 1 week after modeling completion, the mice in the experimental group still showed decreased body weight, but at 2 weeks after modeling completion, no significant differences in body weight were detected among the 3 groups (Fig. 1B).

We analyzed the bacterial diversity of the colon at 72 h after cefoperazone sodium and sulbactam sodium solution gavage. The complexity of species diversity in the intestinal microbial community was analyzed using R language software. The Chao1, observed species, ACE, Simpson, Shannon, and Good coverage indexes in the experimental group significantly differed from those in the control and normal groups (Figure S1).

### *C. Jejuni* colonization in mouse intestines

**Fig. 2:**
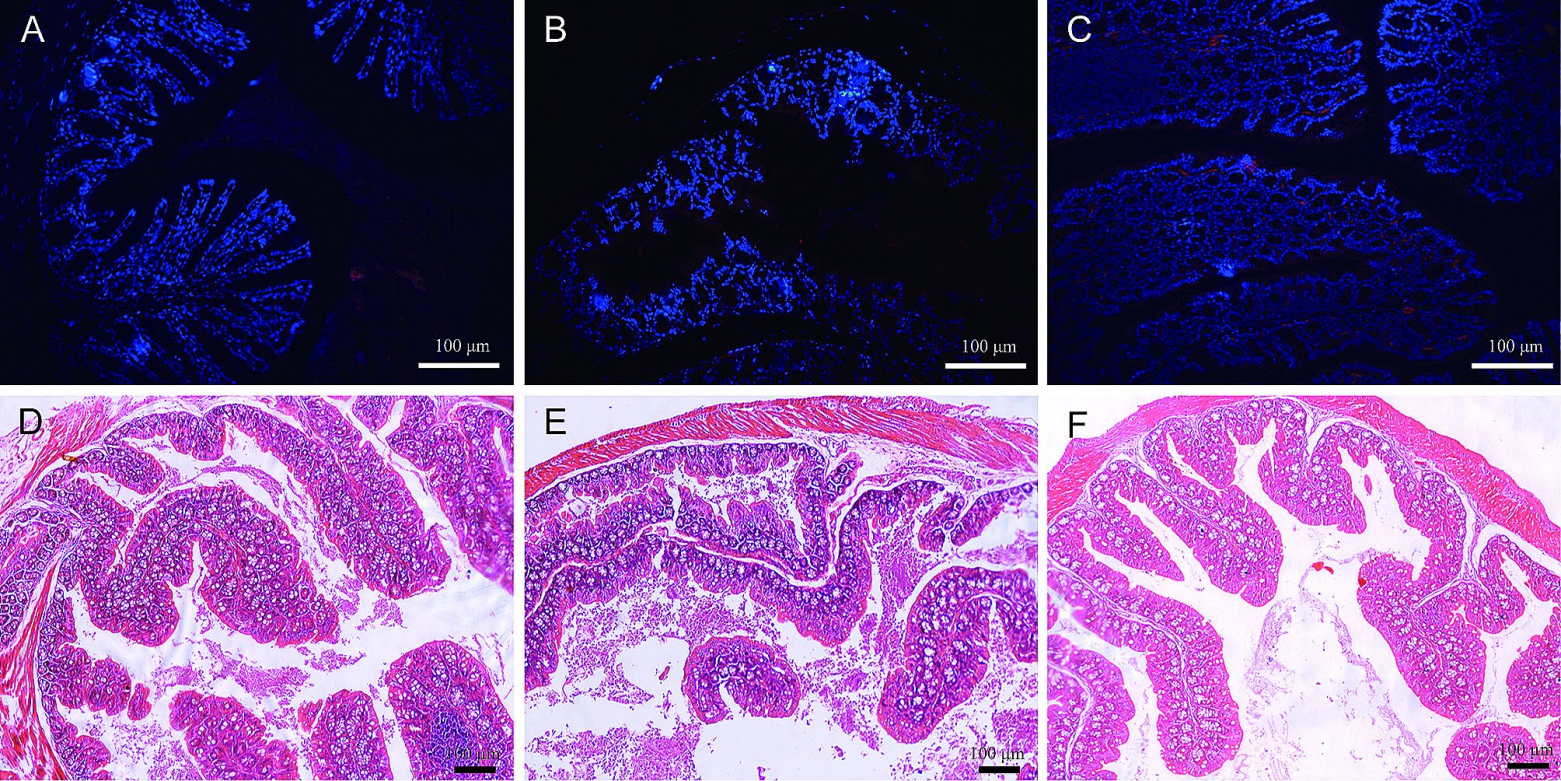
Immunofluorescence and hematoxylin and eosin (HE) staining of mouse intestinal tissues on day 14 after modeling completion. **(A) D.** Normal group. **(B) E.** Control group. **(C) F.** Experimental group. The red fluorescence is *C. jejuni*; the blue is DAPI staining. Scale bar: 100 μm

Immunofluorescence staining for *C. jejuni* (red) was performed on colon tissues harvested from the mice on day 14 after modeling completion. The results showed that on day 14, obvious red fluorescent-labeled *C. jejuni* could be seen in the colonic lumen in the experimental group (Fig. 2C), while no obvious red fluorescent markers were found in the intestinal tracts of the normal (Fig. 2A) and control mice (Fig. 2B). We next determined if *C. jejuni* colonization was associated with intestinal inflammation in the mice. Hematoxylin and eosin staining of the colon tissues harvested on the 7th (Figure S2) and 14th day (Fig. 2D and F) after modeling showed that the intestinal mucosa was mostly intact in the experimental group, with no significant inflammatory-cell infiltration.

### Effects of *C. Jejuni* colonization on the diversity of colonic microbiota in mice

**Fig. 3:**
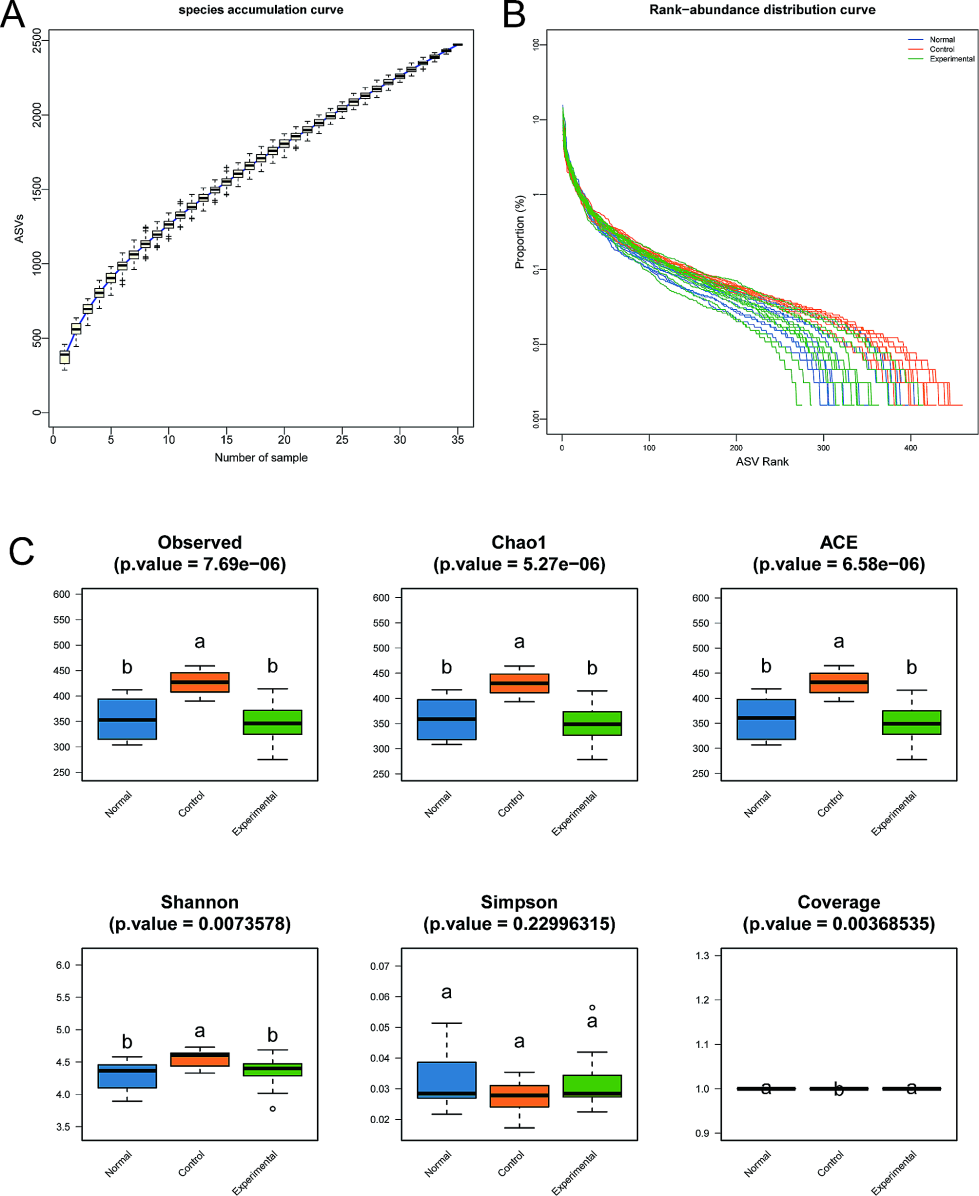
The diversity of the colonic microbiota in the 3 groups of mice, as analyzed using the 16S rDNA method. **(A)** Species accumulation curve. **(B)** Species accumulation box plot. **(C)** Alpha diversity of colonic microbiota, including the Chao1, observed species, abundance-based coverage estimators (ACE), Simpson, Shannon, and Good coverage indexes. ^a^ indicates averages with no significant differences. ^b^ indicates averages with significant differences

In this study, the dilution curve and hierarchical clustering curve were flat and smooth, and the species accumulation box plot initially showed an increasing trend and then stabilized, which suggested that the sample size was sufficient and the results were reliable (Fig. 3A and B).

The complexity of species diversity in the intestinal microbial community was analyzed using R language software. The Chao1, observed species, Simpson, Shannon, and Good coverage indexes did not significantly differ between the experimental and normal groups. In contrast, significant differences in the Chao1 (*P* = 5.27 × 10^− 6^), observed species (*P* = 7.69 × 10^− 6^), Shannon (*P* = 0.0074), and Good coverage indexes (*P* = 0.0037) were found between the control and normal groups (Fig. 3C).

**Fig. 4:**
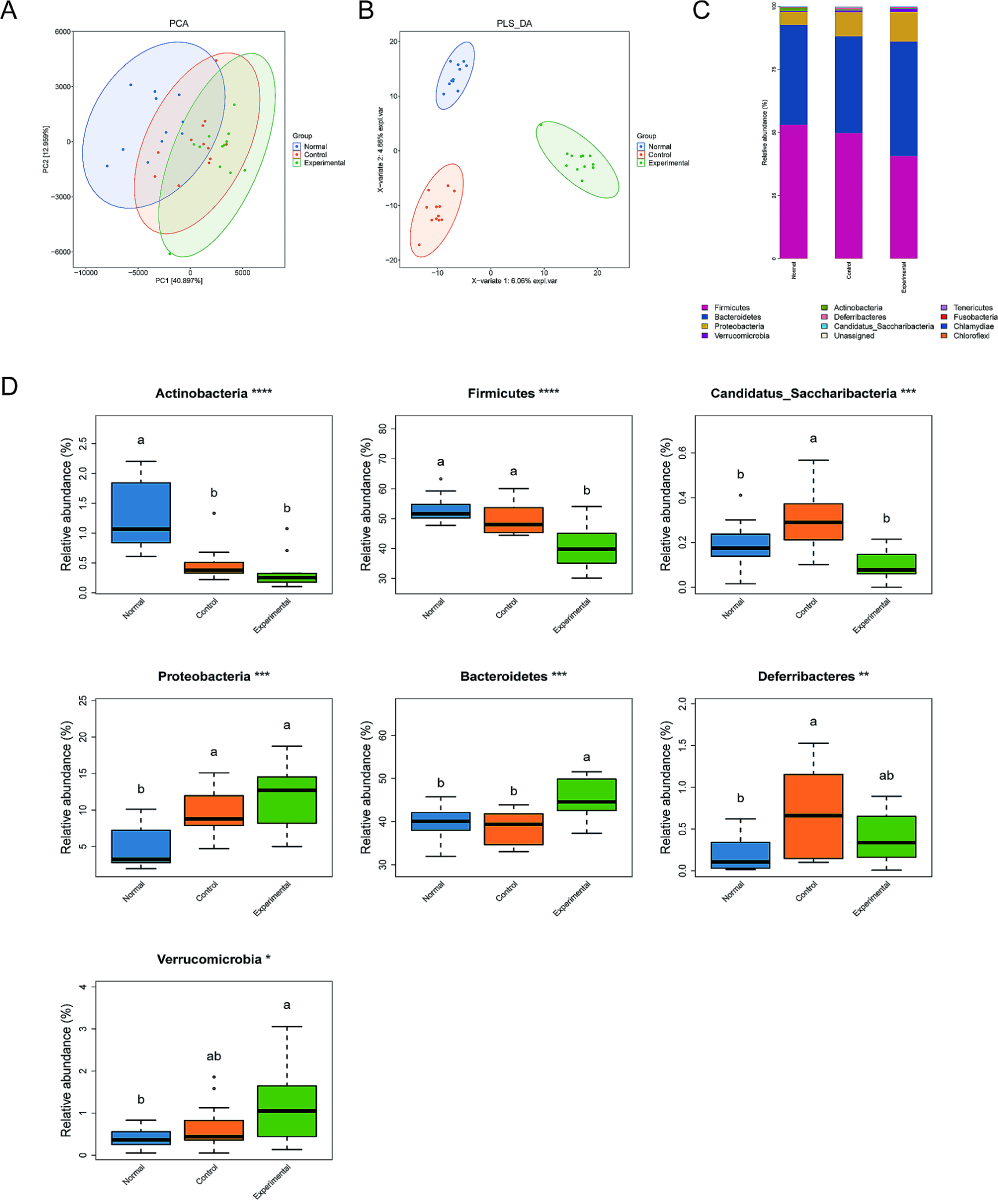
Composition of the colonic microbiota in the 3 groups of mice, as analyzed using the 16S rDNA method. **(A)** Principal component analysis map of the colonic microbiota. **(B)** Partial least squares discriminant analysis of the colonic microbiota. **(C)** Relative abundances of colonic bacteria at the phylum level in the 3 groups. **(D)** Relative abundances of differentially abundant bacterial phyla (Actinobacteria, Firmicutes, Candidatus, Saccharibacteria, Proteobacteria, Bacteroidetes, Deferribacteres, and Verrucomicrobia). ^a^Indicates averages with no significant differences. ^b^Indicates averages with significant differences

Using the OTU/amplicon sequence variant (ASV) abundance table, we screened and visualized the OTUs and ASVs that were unique to each group as well as those that were common between the groups. Principal component analysis was performed using the OTU/ASV abundance table, and variance decomposition was used to reflect the differences between samples on a two-dimensional coordinate map. Samples with similar compositions were clustered together on the map, while samples with different compositions exhibited a dispersed distribution (Fig. 4A). According to the partial least squares discriminant analysis, significant differences were present among the 3 groups (Fig. 4B).

### Effects of *C. Jejuni* colonization on the composition of colonic microbiota in mice

We visualized the bacterial composition and relative abundance of the colonic microbiota by using bar plots, which showed that overall, the following phyla were dominant in the mouse colonic microbiota in all groups: Firmicutes, Bacteroidetes, Proteobacteria, Verrucomicrobia, Actinobacteria, Candidatus, Saccharibacteria, and Deferribacteres (Fig. 4C). In the experimental group, the composition of the colonic microbiota at the phylum level differed from that in the normal and control groups, with the experimental group showing a higher proportion of Bacteroidetes and a lower proportion of Firmicutes (Fig. 4D).

**Fig. 5:**
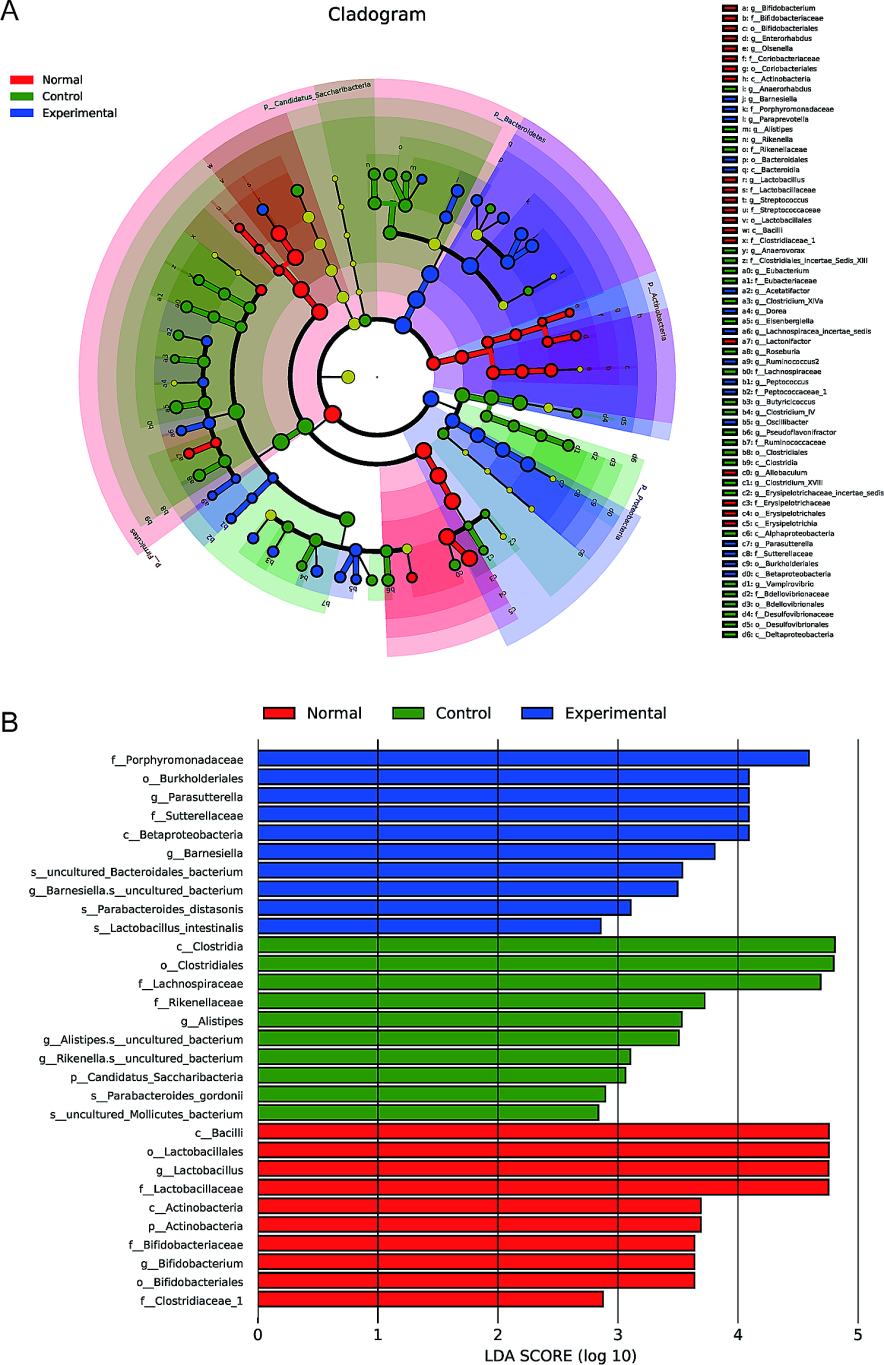
Relative abundances of the major taxa, from the phylum to the genus level, in the 3 groups of mice. **(A)** Cladogram of the colonic bacterial community plotted from the linear discriminant analysis (LDA) effect size (LEfSe) analysis. **(B)** Composition of the colonic microbiota according to the LDA scores

Linear discriminant analysis (LDA) effect size analyses and LDA scores were used to identify the taxa at the phylum and genus levels that showed significantly differential abundance in the mouse colonic microbiota between the groups (Fig. 5). The data showed that Porphyromonadaceae (Bacteroidales), Burkholderiales, *Parasutterella*, *Sutterella*, and Betaproteobacteria were dominant in the experimental group, while Bacilli, Lactobacillales, *Lactobacillus*, Lactobacillaceae, and Actinobacteria were dominant in the normal group. Clostridia, Clostridiales, Lachnospiraceae, Rikenellaceae, and *Alistipes* were dominant in the control group.

## Discussion

*C. jejuni* has a wide range of hosts, including birds and mammals; it is one of the most important pathogenic bacteria in the genus *Campylobacter*, and causes gastroenteritis in humans worldwide. However, *C. jejuni* is unable to colonize the digestive tract of mice, and the intestinal microbiota of mice is considered to be an important factor that prevents *C. jejuni* colonization. In this study, cefoperazone sodium and sulbactam sodium were continuously administered by gavage for 2 days to induce depletion of the intestinal microbiome in mice, causing an imbalance of the intestinal microbiota [18]. This allowed *C. jejuni* to colonize the intestinal tract of the mice. Bacterial community composition analysis of the intestinal contents showed that *Lactobacillus* in the ileum and *Bacteroides* in the caecum and colon may be important bacterial groups that provide resistance to colonization by pathogens [19, 20].

It is generally believed that the presence of *C. jejuni* in fresh feces or its direct culture from animal intestines is indicative of successful colonization of the digestive tract. In this study, *C. jejuni* was detected in the feces of mice 14 days after modeling. Additionally, immunofluorescence staining of intestinal tissue sections confirmed that *C. jejuni* had colonized the mouse gut from day 7 to day 14 after modeling completion; however, no obvious inflammation was observed on histopathological examination, suggesting that *C. jejuni* may not have entered deep into the intestinal tissue.

To colonize the surface of the jejunal and ileal mucosal cells, *C. jejuni* relies on the directional chemotactic movement of its flagella through the mucus layer on the mucosal surface. The chemotaxis protein CheY is responsible for transmitting sensory signals from the chemoreceptors of *C. jejuni* to its flagella and regulating the clockwise rotation of the flagella. Studies have shown that after the inactivation of CheY in *C. jejuni*, motility and invasion are not altered, but chemotaxis is lost, resulting in an inability to colonize intestinal cells in animals [21, 22]. Liu Shuo [23] challenged Kunming mice by oral gavage with the *C. jejuni lulei* strain, and collected their feces to isolate and culture *C. jejuni*. The isolated strains were then used to challenge a new batch of mice and were serially passaged for 18 generations, resulting in enhanced virulence and stable genetic isolates. The strain used in the present study is the quality control standard strain ATCC33291 of *C. jejuni*. However, if other invasive or more pathogenic strains are used, different experimental results may be observed.

The present study revealed that *C. jejuni* colonization significantly altered the diversity of the colonic bacterial community in mice and caused taxonomic changes in the colonic microbiota. Although the experimental group underwent microbiota depletion followed by *C. jejuni* infection, while the control group underwent only *C. jejuni* infection, it is imperative to consider the dynamic nature of microbial ecosystems [24, 25]. One plausible explanation for the higher species diversity in the control group, in comparison to both the normal and experimental groups, may involve the presence of pre-existing colonization-resistance mechanisms within the control group. These mechanisms potentially facilitated the proliferation of other microbial species, consequently enhancing diversity and fostering the establishment of a more stable and diversified microbial community in the short term [26]. Conversely, deliberate microbiota depletion prior to *C. jejuni* infection in the experimental group likely contributed to a transient reduction in species diversity. This reduction could be attributed to the recovery of the microbial community from the depletion process and its subsequent adaptation to the presence of the pathogen. Consequently, this transient disruption might have resulted in a lower diversity than that in the control group during the initial stages of infection. Overall, while the observed discrepancy in species diversity among the groups is atypical, it underscores the intricate dynamics of microbial communities in response to perturbations such as microbiota depletion and pathogen invasion.

Specifically, the intestinal microbiota exhibited increased and decreased relative abundances of Bacteroidetes and Firmicutes, respectively. In humans, Bacteroidetes participates in several important metabolic processes in the colon, such as the fermentation of carbohydrates, utilization of nitrogen-containing substances, and biotransformation of bile acids and other steroids [27]. Most intestinal bacteria are glycolytic, meaning that they obtain energy and carbon through the carbohydrate hydrolysis. Bacteroidetes and Firmicutes are two of the main intestinal flora; they have a symbiotic relationship with the host, and can jointly promote energy absorption or storage by the host. Not only are Bacteroidetes and Firmicutes crucial for the fermentation of polysaccharides in the digestive tract, the proportion of the two is also important [28]. Decreased Bacteroidetes abundance or increased Firmicutes abundance can promote obesity. In the current study, only the weight of the mice was monitored during the experiment, and no significant changes were observed. However, it would be interesting to investigate whether the variation in the intestinal microbiota caused by *C. jejuni* colonization induces obesity in mice.

The digestive tract contains a complex immune system that includes immune cells, immune tissues, and immune action factors, which eliminate invading pathogenic bacteria through sustained and effective interactions [29]. Changes in the immune system of the mice can allow *C. jejuni* to colonize their intestines. Some researchers have administered broad-spectrum antibiotics such as oral ampicillin and vancomycin to immunodeficient transgenic mice before subjecting the mice to *C. jejuni* infection. The results showed that *C. jejuni* could colonize the colon, mesenteric lymph nodes, and spleen after antibiotic exposure [8, 17, 30]. Moreover, *C. jejuni* can effectively colonize the gut of germ-free mice and spread to their immune tissues and organs [31]. However, immune gene-deficient mice and germ-free mice exhibit significant changes in their immune systems, and cannot effectively simulate *C. jejuni* infection in the intestinal tract of healthy people nor the subsequent *C. jejuni*-induced diseases. Furthermore, recent high-throughput gut metagenomic sequencing analysis has revealed that gender has the greatest impact on the gut microbiota [32, 33]. In this study, to maintain relatively consistent gut microbiota, mice in the same group were kept in the same cage for a feeding period longer than 2 weeks, and female mice were selected. Thus, it remains uncertain whether gender or hormones affect the colonization of *C. jejuni*, and this topic requires further investigation.

## Conclusions

In conclusion, this study confirmed that microbiome depletion induced by cefoperazone sodium and sulbactam sodium can promote long-term colonization of *C. jejuni* in the mouse gut. However, further research is needed to create a mouse model of *C. jejuni* infection.

### Electronic supplementary material

Below is the link to the electronic supplementary material.

Supplementary Material 1

## Data Availability

The raw sequence data reported in this paper have been deposited in the Genome Sequence Archive (Genomics, Proteomics & Bioinformatics 2021) in National Genomics Data Center (Nucleic Acids Res 2022), China National Center for Bioinformation / Beijing Institute of Genomics, Chinese Academy of Sciences (GSA: CRA013946) that are publicly accessible at https://ngdc.cncb.ac.cn/gsa.

## Abbreviations

*C. jejuni*: *Campylobacter jejuni*
*HipO*: hippurate hydrolase
qPCR: quantitative polymerase chain reaction
GBS: Guillain-Barré syndrome
DAPI: 4’,6-diamidino-2-phenylindole
OUT: operational taxonomic unit
RDP: Ribosomal Database Project
ASV: amplicon sequence variant
LDA: Linear discriminant analysis
