# Supplementary material for: Antibiotic-induced microbiome depletion promotes intestinal colonization by Campylobacter jejuni in mice
Source: BMC Microbiol. 2024 May 9;24:156. doi: 10.1186/s12866-024-03313-5 (PMC11080253; doi:10.1186/s12866-024-03313-5)
Supplement: Supplementary file 1 — Supplementary Material 1 [file 12866_2024_3313_MOESM1_ESM.docx]

**Supplementary materials**

**
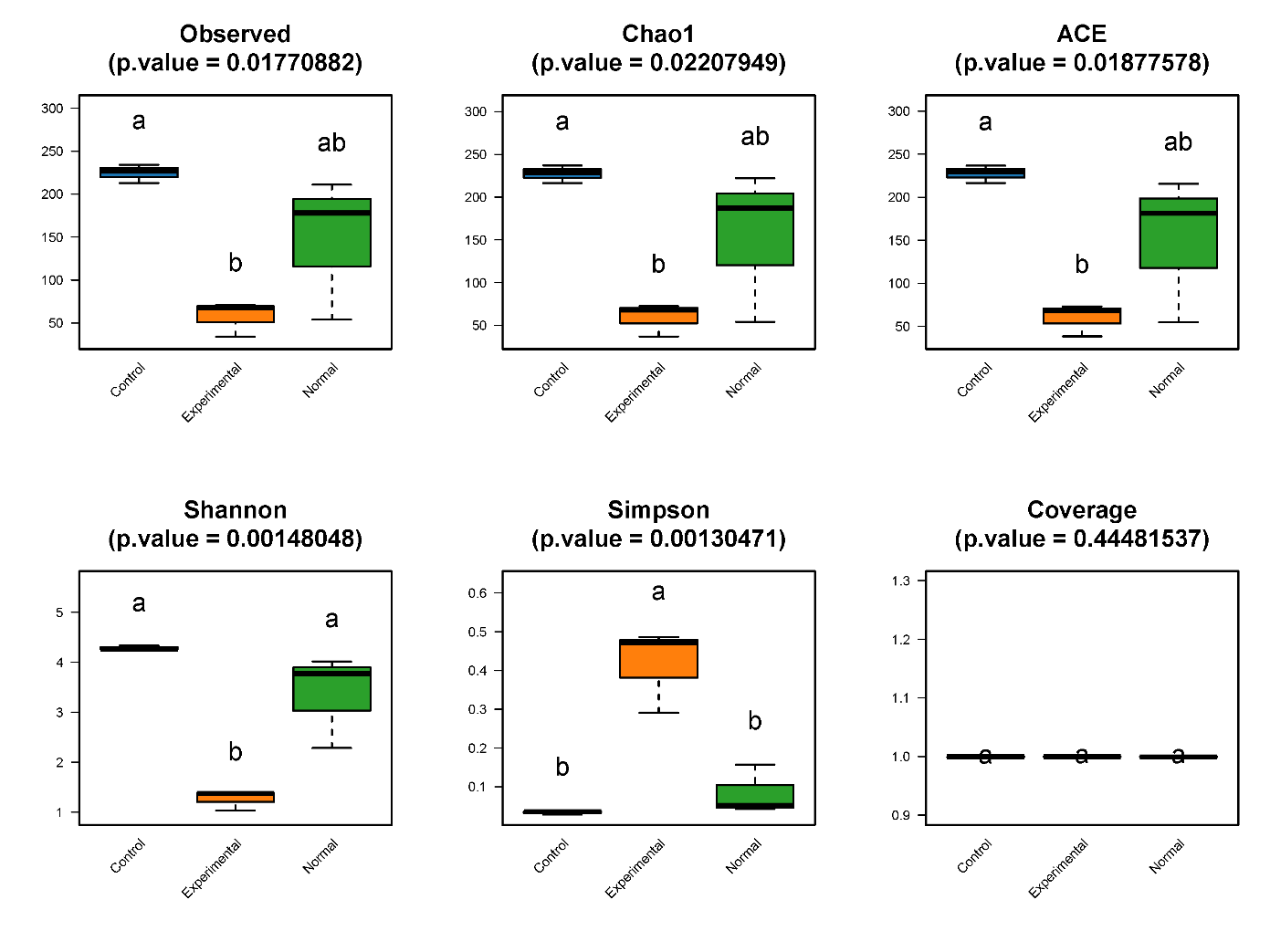
**

**Figure S1.** Composition of the colonic microbiota at 72 h after cefoperazone sodium and sulbactam sodium solution gavage, as analyzed using the 16S rDNA method.


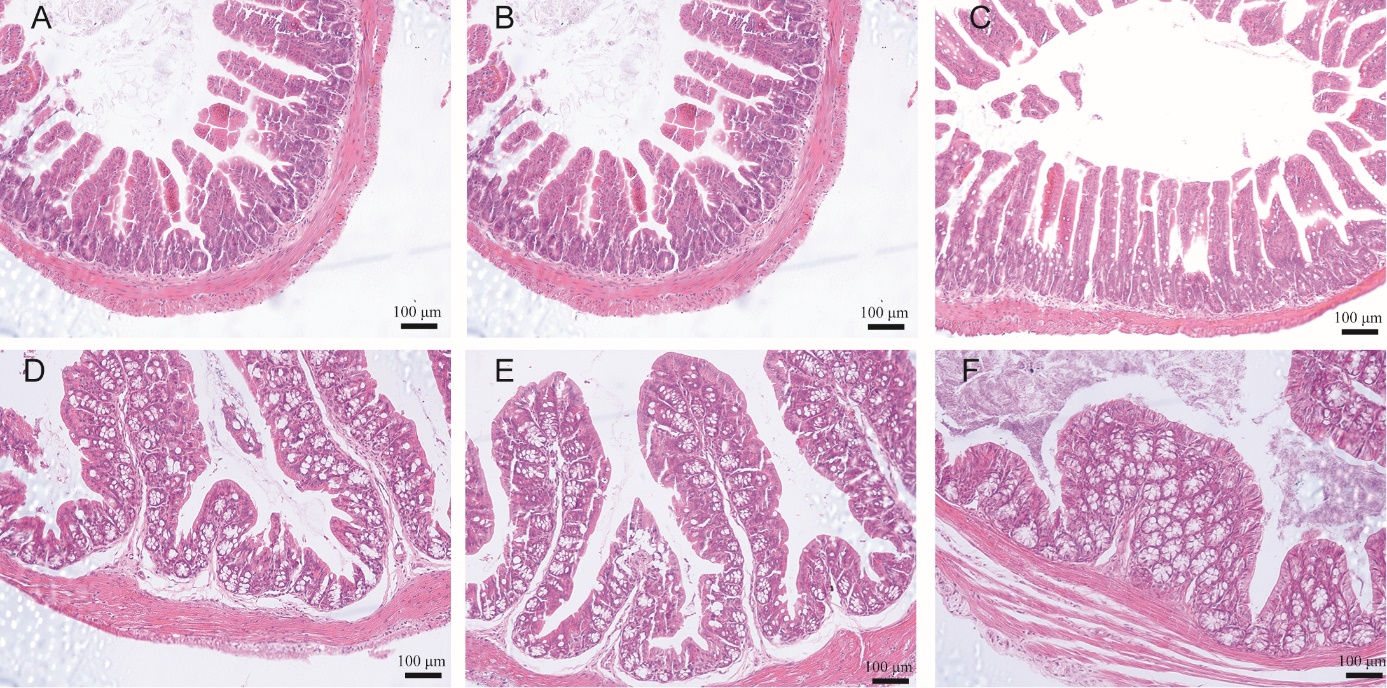


**Figure S2.** Hematoxylin and eosin (HE) staining of mouse intestinal tissues on day 7 after modeling completion. **A.** Ileum tissue in the normal group. **B.** Ileum tissue in the control group. **C.** Ileum tissue in the experimental group. **D.** Colon tissue in the normal group. **E.** Colon tissue in the control group. **F.** Colon tissue in the experimental group. Scale bar: 100 μm.
